# Repeated Low-Level Blast Overpressure Leads to Endovascular Disruption and Alterations in TDP-43 and Piezo2 in a Rat Model of Blast TBI

**DOI:** 10.3389/fneur.2019.00766

**Published:** 2019-07-30

**Authors:** Lanier Heyburn, Rania Abutarboush, Samantha Goodrich, Rodrigo Urioste, Andrew Batuure, Jonathan Statz, Donna Wilder, Stephen T. Ahlers, Joseph B. Long, Venkata Siva Sai Sujith Sajja

**Affiliations:** ^1^Walter Reed Army Institute of Research, Blast Induced Neurotrauma Branch, Silver Spring, MD, United States; ^2^Neurotrauma Department, Naval Medical Research Center, Silver Spring, MD, United States; ^3^Henry M. Jackson Foundation for the Advancement of Military Medicine, Inc., Bethesda, MD, United States

**Keywords:** blast-induced neurotrauma, blood-brain barrier, Piezo2, TDP-43, low-level blast, training relevant, repeated exposures, cumulative effects

## Abstract

Recent evidence linking repeated low-level blast overpressure exposure in operational and training environments with neurocognitive decline, neuroinflammation, and neurodegenerative processes has prompted concern over the cumulative deleterious effects of repeated blast exposure on the brains of service members. Repetitive exposure to low-level primary blast may cause symptoms (subclinical) similar to those seen in mild traumatic brain injury (TBI), with progressive vascular and cellular changes, which could contribute to neurodegeneration. At the cellular level, the mechanical force associated with blast exposure can cause cellular perturbations in the brain, leading to secondary injury. To examine the cumulative effects of repetitive blast on the brain, an advanced blast simulator (ABS) was used to closely mimic “free-field” blast. Rats were exposed to 1–4 daily blasts (one blast per day, separated by 24 h) at 13, 16, or 19 psi peak incident pressures with a positive duration of 4–5 ms, either in a transverse or longitudinal orientation. Blood-brain barrier (BBB) markers (vascular endothelial growth factor (VEGF), occludin, and claudin-5), transactive response DNA binding protein (TDP-43), and the mechanosensitive channel Piezo2 were measured following blast exposure. Changes in expression of VEGF, occludin, and claudin-5 after repeated blast exposure indicate alterations in the BBB, which has been shown to be disrupted following TBI. TDP-43 is very tightly regulated in the brain and altered expression of TDP-43 is found in clinically-diagnosed TBI patients. TDP-43 levels were differentially affected by the number and magnitude of blast exposures, decreasing after 2 exposures, but increasing following a greater number of exposures at various intensities. Lastly, Piezo2 has been shown to be dysregulated following blast exposure and was here observed to increase after multiple blasts of moderate magnitude, indicating that blast may cause a change in sensitivity to mechanical stimuli in the brain and may contribute to cellular injury. These findings reveal that cumulative effects of repeated exposures to blast can lead to pathophysiological changes in the brain, demonstrating a possible link between blast injury and neurodegenerative disease, which is an important first step in understanding how to prevent these diseases in soldiers exposed to blast.

## Introduction

According to the U.S. Department of Defense, traumatic brain injury (TBI) was reported in ~380,000 service members between 2000 and 2017, and 82.3% of these TBIs were classified as mild (mTBI) (1). Due to the widespread use of improvised explosive devices and other explosives in more recent military conflicts, blast-related mTBI has emerged as one of the most common types of injuries sustained by warfighters (2, 3). In addition to blast injury sustained during combat, military, and law enforcement personnel who participate in training with explosives, such as breaching and heavy weapon systems, are exposed to multiple low-level blast exposures throughout training (4). The instructors for these exercises are cumulatively exposed to many more low-level blasts over a longer period of time throughout their careers (4). “Safe levels” of blast are loosely based upon exposures not exceeding pressures causing tympanic membrane rupture, which occurs at 4 psi blast levels for an unprotected ear (3). However, blast exposures experienced by Warfighters in operational settings during breaching, firing heavy weapon systems, or by explosive ordinance personnel in theater, could far exceed 13 psi static levels due to the close proximity to these blasts compared to what is experienced in training. These situations have prompted growing concern over Warfighters' well-ness for operational readiness in the short term and continued operational readiness in the mid-term (4).

There is evidence that repetitive blast exposure leads to acute and chronic symptoms that mimic those seen following mTBI. A study in which about half of participants had more than 5 years breaching experience showed that low-level blast exposure can lead to symptomatology similar to that seen in mTBI including headache, irritability, and cognitive deficits (5). A tremendous amount of pre-clinical research has been conducted to understand the effects of single blast exposure on the brain at various magnitudes of pressure, showing neuronal loss, cell death, blood- brain barrier (BBB) disruption, astroglial activation, and behavioral changes (6–9). While there is a growing concern that repeated low-level blast exposure is experienced by the Warfighter, research to understand the biological changes occurring in the brain is currently lacking. Repeated low-level exposures may prompt subtle behavioral and pathological changes that can result from altered BBB permeability, pathological protein accumulation, and disruptions in pressure-sensing mechanisms in the brain, without obvious anatomical changes (10). The relationship between repetitive blast exposure interval/frequency and brain pathology is currently unclear.

The neurological and neurocognitive changes experienced by those who have sustained multiple blast exposures may have many different cellular and anatomical underpinnings compared to those who have sustained clinically-diagnosed concussion. Of interest in this study, and a prominent feature of neurotrauma is alteration of the blood-brain barrier (BBB), a semi-permeable border separating the cerebral circulation from the brain parenchyma formed mainly by tight junction proteins (occludin and claudin-5) between endothelial cells lining the vasculature. Formation of new blood vessels in the brain, angiogenesis, is mediated by vascular endothelial growth factor (VEGF), which can contribute to BBB opening and increased vulnerability to injury of the central nervous system (11–13). Also of interest is alteration in the transactive response DNA binding protein 43 kDa (TDP-43), which is found to abnormally accumulate in a variety of neurodegenerative diseases as well as after brain trauma (14, 15). We also examined a mechanosensitive transmembrane protein called piezo2, which functions as a pressure sensor, opening a cation channel in response to mechanical and pressure changes and causing downstream cellular changes (16–19).

Due to the high incidence of low-level blast exposure in military personnel and in related training, it is important to understand how repeated low-level blast exposure can lead to acute and potentially chronic neurological deficits. In this study, an advanced blast simulator (ABS), which closely mimics “free-field” blast, was used to expose rats to multiple blasts at varying intensities. Brains were evaluated for alterations in the expression of TDP-43 and piezo2 as well as disruptions or changes in blood brain barrier integrity (claudin-5, occluding, and VEGF) at an acute term to study the immediate effects of blast.

## Materials and Methods

### Animals

All animal experiments were conducted in accordance with the Animal Welfare Act and other federal statutes and regulations relating to animals and experiments involving animals, and adhered to principles stated in the Guide for the Care and Use of Laboratory Animals (NRC Publication 2011 edition) using an Institutional Animal Care and Use Committee approved protocol. Male Sprague Dawley rats, 8–9 weeks old (*n* = 6 per group) that weighed ~275 g (Charles River Laboratories, Wilmington, MA) were housed at 20–22°C (12 h light/dark cycle) with free access to food and water *ad libitum*.

### Blast Overpressure Exposure

**Figure 1:**
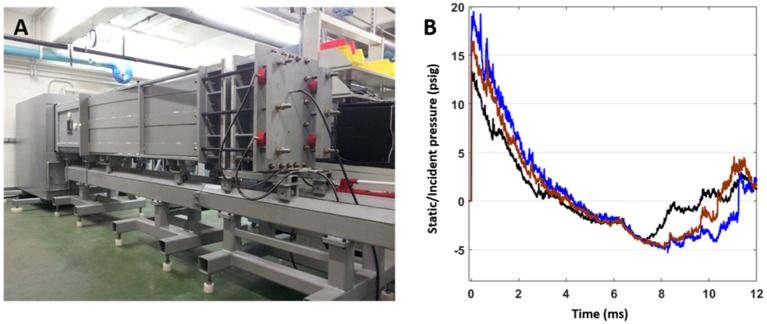
**(A)** The advanced blast simulator (ABS) located at Walter Reed Army Institute of Research (WRAIR), used to produce experimental blast. **(B)** Pressure profiles generated using the ABS, which has both positive and negative phases and mimics “free-field” blast for the 13 (black), 16 (brown), and 19 psi (blue) treatment groups.

Rats were anesthetized with isoflurane and subjected to survivable blast overpressures using an ABS located at the Walter Reed Army Institute of Research (WRAIR). The ABS consists of a 0.5 ft long compression chamber that is separated from a 21 ft long transition/expansion test section (Figure 1). The anesthetized rat was secured in the test section in a longitudinal (head-on; on-axis) or transverse (side-on; off-axis) orientation to the direction of blast exposure. The compression chamber was pressurized with room air, causing membranes to rupture at a pressure that is dependent upon the thickness of the specific membrane sheet separating the two chambers, yielding a supersonic blast wave that impacts the experimental subject in the test section. To yield a range of mild to moderate TBI in rats in these experiments, Valmex® membranes (Mehler texnologies, VA) were used to yield peak positive static pressures of 13 (impulse: 17.27 ± 0.51 psi^*^ms), 16 (impulse: 23.99 ± 0.51 psi^*^ms), and 19 psi (impulse: 29.87 ± 0.51 psi^*^ms) with a positive phase duration of 4–5 ms (Table 1) with a negative peak static pressures of 3.96 ± 0.11, 4.61 ± 0.18, and 4.76 ± 0.19 psi, respectively (Table 1). Animals (*n* = 6 per group) were exposed to a single daily blast of 13, 16, or 19 psi for either 1 (1x), 2 (2x), 3 (3x), or 4 days (4x) from the front or from the side; repeated blast exposures were separated by 24 h. Animals were randomly assigned to different blast exposure groups or the sham group to reduce variability's from unforeseen confounding factors and ensure that any observed differences can be attributed to the blast regimen. Animals were blasted between 9 and 11 a.m. in a temperature- and humidity-controlled room by the same technicians each time. All sham animals were subjected to isoflurane anesthesia, loading in the shock tube, and recovery procedures as described above, but were not exposed to blast overpressure (BOP). At 24 h following final BOP exposure, animals were euthanized with isoflurane overdose and whole hemisphere brain tissue was flash frozen on dry-ice and stored at −80°C until further analysis.

**Table 1:** Pressure profiles of 13, 16, and 19 psi treatment groups, including: peak positive static pressure, peak positive dynamic pressure, peak negative static pressure, impulse positive static pressure, impulse positive dynamic pressure, impulse negative static pressure, and duration.

**Group**	**Pressure type**		**Peak (psi)**	Impulse (psi*ms)	**Duration (ms)**
13 psi	Positive phase	Static	13.06 ± 0.39	17.27 ± 0.51	3.75
		Dynamic	8.31 ± 0.33	8.97 ± 0.62	
	Negative phase	Static	3.96 ± 0.11	11.37 ± 0.36	5.87
16 psi	Positive phase	Static	16.14 ± 0.48	23.99 ± 0.51	4.11
		Dynamic	11.56 ± 0.58	14.38 ± 0.86	
	Negative phase	Static	4.61 ± 0.18	16.92 ± 0.51	6.25
19 psi	Positive phase	Static	19.10 ± 0.48	29.87 ± 0.51	4.27
		Dynamic	13.31 ± 0.90	19.68 ± 1.40	
	Negative phase	Static	4.76 ± 0.19	19.08 ± 0.51	7.14

### Protein Extraction

After euthanasia, the right cerebrum was homogenized at 5% w/v in T-PER Tissue Protein Extraction Reagent (cat# 78510, ThermoFisher, NY) with 1% protease/phosphatase inhibitor cocktail (Sigma-Aldrich). Homogenate was centrifuged at 5000 x g for 5 min at 4°C. The supernatant, containing soluble protein fraction was collected and stored at −80°C until use for biochemical assays (Western blot, ELISA).

### ELISA

Due to concern that VEGF changes would be relatively small and not readily detectable by Western blots, a more sensitive test with higher throughput was used to measure VEGF. Specifically, a rat VEGF ELISA kit was used to measure levels of VEGF in the brain (cat# RAB0512, Sigma-Aldrich). The ELISA was run in triplicate on one plate in order to capture these expected small changes. All samples were triplicated and assay was run according to manufacturer's instruction. Samples and standards were loaded into wells, covered, and incubated for 2.5 h at room temperature. The wells were washed 4 times with 1x Wash Solution. Hundred micro liter of 1x Biotinylated Detection Antibody was added to each well for 1 h at room temperature with gentle shaking. Wells were washed 4 times with 1x Wash Solution. Hundred micro liter of prepared HRP-Streptavidin solution was added to each well and incubated for 45 min at room temperature with gentle shaking. Wells were washed 4 times with 1x Wash Solution. Hundred micro liter ELISA Colorimetric TMB Reagent was added to each well and incubated for 30 min at room temperature with gentle shaking. Fifty microliter of Stop Solution was added to each well to stop the reaction and the plate was immediately read at 450 nm using SpectraMax M5 with SoftMax 5.2 software (Molecular Devices). VEGF levels were calculated as protein concentration based on standard curve.

### Western Blot

Western blot samples were prepared by mixing sample homogenates (~25 μg) with buffer containing loading dye to a volume of 20 μl, which was loaded into a NuPAGE™ 4–12% 1.0 mm, 12-well Bis-Tris Protein Gel (cat# NP0322BOX, ThermoFisher). Gels were run at 180 V for 35 min. Separated protein products were transferred to a PVDF membrane using the iBlot PVDF Transfer Stack and iBlot2 Dry Blotting System (ThermoFisher). The membranes containing protein products were blocked for non-specific binding for 1 h at room temperature and were then incubated overnight at 4°C in primary antibody. After several washes, the membranes were then incubated for 1 h at room temperature in HRP-linked secondary antibody. Membranes were washed again before developing. Membranes were incubated for 5 min at room temperature in Pierce ECL Western Blotting Substrate (ThermoFisher) and were imaged using a FluorChem HD2 imager (Protein Simple). Bands were analyzed by densitometry analysis using ImageJ software (NIH) and protein levels were determined using β-actin as a loading control, which did not show any variability between injury conditions. Primary antibodies: rabbit polyclonal antibody against TDP-43 (1:2,000, ProteinTech cat# 10782-2-AP), rabbit polyclonal antibody against FAM38B/Piezo2 (1:2,000, ProSci cat# 26-438), mouse monoclonal antibody against Occludin (1:2,000, ThermoFisher cat# 33-1,500), mouse monoclonal antibody against Claudin-5 (1:2000, ThermoFisher cat# 35-2,500), and mouse monoclonal antibody against β-actin (1:20,000 abcam A2228). Secondary HRP antibodies against rabbit (1:2,500, cat# 65-6120) and mouse (1:2,500, Thermo cat# 32430).

### Statistical Analysis

There were no significant differences found between sham groups (e.g., 1 vs. 4x sham), therefore all sham data was combined and comparisons were made between experimental groups vs. pooled shams. The non-parametric Kruskal-Wallis test was performed to and for each protein, it was determined that there were significant differences between groups (*p* < 0.001). Therefore, the non-parametric Mann-Whitney *U*-test was performed to compare values of experimental groups vs. shams. Corrections for multiple comparisons were performed and adjusted *p*-values for the actual number of comparisons of interest (not every data group was compared to every other data group) were used where appropriate. A significance level of *p* < 0.05 was considered statistically significant. Unless otherwise specified, all data are expressed as mean ± SEM.

## Results

### VEGF

**Figure 2:**
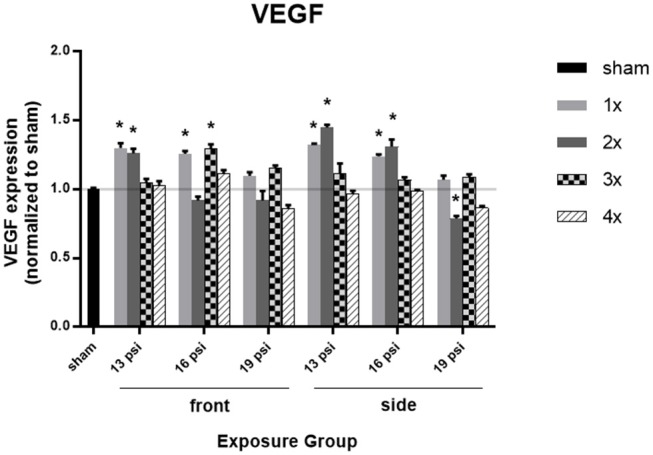
Quantification of VEGF expression measured by ELISA. Results presented as blast intensity for each orientation (front vs. side). Statistical significance compared to sham: ^*^p < 0.05 (Significance values in Supplemental Table 1). Data is expressed as mean ± SEM, normalized to sham.

To determine the effect of BOP on brain vascularization, we measured expression of VEGF in the brain via ELISA. Overall, levels of VEGF were increased after 16 psi BOP exposure, but not after 19 psi BOP exposure. Animals exposed to 19 psi BOP in the frontal position did not have any significant changes in VEGF expression (Figure 2). Animals exposed to 19 psi BOP from the side did show a significant decrease in VEGF after 2x blast exposures (~21%), but no changes were observed after 1x, 3x, or 4x exposures at 19 psi (Figure 2). Animals exposed to 16 psi BOP from the front had a significant increase in VEGF after 1x (~26%), and 3x exposures (~29%), with no significant changes after 2x or 4x blasts. Animals exposed to 16 psi BOP from the side had significantly increased VEGF after 1x (~24%) and 2x (~31%) exposures, but not after 3x or 4x. Finally, animals exposed to 13 psi BOP from the front had a significant increase in VEGF after 1x (~30%) and 2x (~26%) exposures, but not after 3x or 4x. Similarly, animals exposed to 1x (~32%) or 2x (~45%) blasts of 13 psi from the side had significantly increased VEGF, but no change after 3x or 4x exposures.

### Occludin

**Figure 3:**
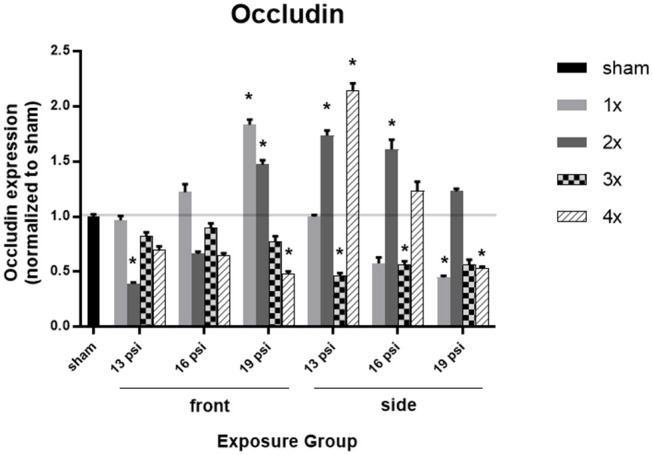
Quantification of occludin expression measured by Western blot. Results presented as blast intensity for each orientation (front vs. side). Values normalized to sham. Statistical significance compared to sham: ^*^p < 0.05 (Significance values in Supplemental Table 2). Data is expressed as mean ± SEM, normalized to sham. Representative Westerns shown in Supplemental Figure 1.

To investigate an underpinning of BBB integrity, we examined the expression of the tight junction protein occludin using Western blot. There was not a uniform pattern in occludin expression following blast exposure, with increased occludin observed in some exposure groups [e.g., 1x 19 (front), 2x 19 (front), 2x 16 (side), 2x 19 (side), and 4x 19 (side)], and decreased occludin in others (Figure 3). Animals exposed to 19 psi BOP from the front had a significant increase in occludin after 1x (~83%) and 2x (~47%) exposures, but a significant decrease after 4x exposures (~52%). Likewise, animals exposed to 19 psi BOP from the side had a significant decrease in occludin after 1x (~55%) and 4x (~47%) exposures and no significant changes after 2 or 3x exposures (Figure 3). Animals exposed to 16 psi BOP from the front did not have any significant changes in occludin, but those exposed to 16 psi from the side had a significant increase after 2x exposures (~61%) and a significant decrease after 3x exposures (~43%). Animals exposed to 13 psi BOP from the front had significantly decreased levels of occludin in the brain after 2x exposures (~61%), but no changes after 1x, 3x, or 4x exposures. Animals exposed to 13 psi BOP from the side had significantly increased levels of occludin after 2x (~74%) and 4x (~115%) exposures, but significantly decreased levels of occludin after 3x exposures (~54%).

### Claudin-5

**Figure 4:**
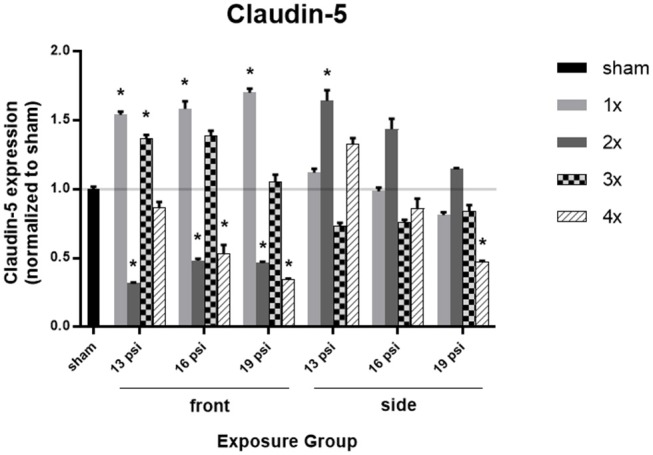
Quantification of claudin-5 expression measured by Western blot. Results presented as blast intensity for each orientation (front vs. side). Values normalized to sham. Statistical significance compared to sham: ^*^p < 0.05 (Significance values in Supplemental Table 3). Data is expressed as mean ± SEM, normalized to sham. Representative Westerns shown in Supplemental Figure 1.

Brain levels of the tight junction protein claudin-5, as measured by Western blot, are illustrated in Figure 4. Animals exposed to 19 psi BOP from the front had significantly increased claudin-5 in the brain after 1x blast exposure (~70%), but significantly decreased claudin-5 after 2x (~54%) and 4x exposures (~66%). Animals exposed to 19 psi BOP from the side had a significant decrease in claudin-5 after 4x exposures (~53%). Animals exposed to 16 psi BOP from the front had a significant increase in claudin-5 after 1x exposure (~58%), but a significant decrease after 2x (~52%) and 4x (~47%) exposures, while those exposed to 16 psi BOP from the side did not have a significant change in claudin-5 expression. Animals exposed to 13 psi BOP from the front had significantly increased claudin-5 after 1x (~54%) and 3x (~37%) exposures, but significantly decreased claudin-5 after 2x (~68%) exposures. Those exposed to 13 psi BOP from the side had a significant increase in claudin-5 after 2x exposures (~64%).

### TDP-43

**Figure 5:**
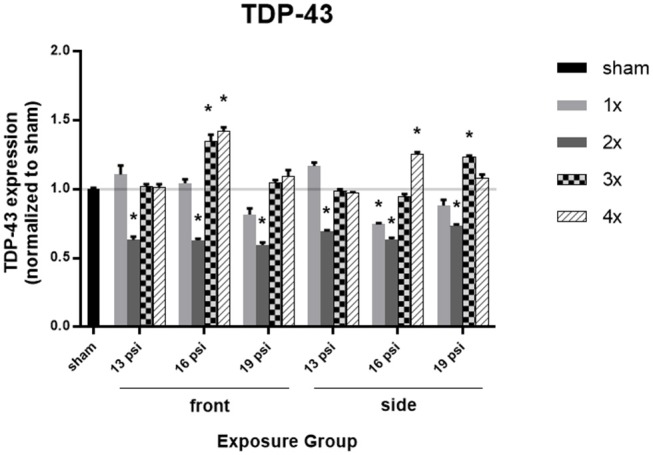
Quantification of TDP-43 expression measured by Western blot. Results presented as blast intensity for each orientation (front vs. side). Values normalized to sham. Statistical significance compared to sham: ^*^p < 0.05 (Significance values in Supplemental Table 4). Data is expressed as mean ± SEM, normalized to sham. Representative Westerns shown in Supplemental Figure 2.

The effect of blast on TDP-43 expression levels, which are normally very tightly regulated in the brain, are shown in Figure 5. There was a significant decrease in TDP-43 expression in all animals after 2x blast exposures, but variations in expression after exposure to other numbers of blast. Animals exposed to 19 psi BOP from the front had a significant decrease in TDP-43 after 2x exposures (~40%), but no change after 1x, 3x, or 4x exposures. Animals exposed to 19 psi BOP from the side had a significant decrease in TDP-43 after 2x exposures (~26%), but a significant increase after 3x exposures (~23%). Animals exposed to 16 psi BOP from the front had a significant decrease in TDP-43 after 2x (~37%) exposures, but significant increases in TDP-43 after 3x (~35%) or 4x (~42%) exposures. Animals exposed to 16 psi BOP from the side also had a significant decrease in TDP-43 after 1x (~25%) and 2x (~36%) exposures, but a significant increase after 4x (~25%) exposures. Finally, there was a significant reduction in TDP-43 after exposure to 2x blasts of 13 psi, from the front (~37%) or side (~31%).

### Piezo2

**Figure 6:**
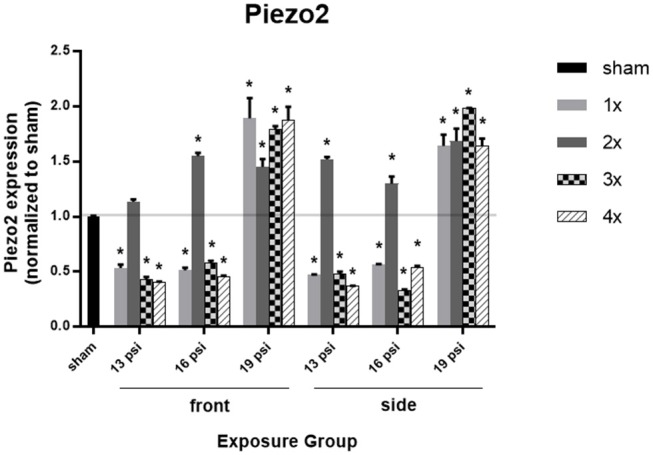
Quantification of Piezo2 expression measured by Western blot. Results presented as blast intensity for each orientation (front vs. side). Values normalized to sham. Statistical significance compared to sham: ^*^p < 0.05 (Significance values in Supplemental Table 5). Data is expressed as mean ± SEM, normalized to sham. Representative Westerns shown in Supplemental Figure 2.

Lastly, we examined the mechanosensitive protein piezo2 using Western blot, to determine whether blast exposure causes alterations in pressure sensitivity (Figure 6). Higher intensity blast (19 psi) caused significant increases in piezo2 expression, while lower intensity blast caused significant decreases in piezo2 expression. Exposure to 19 psi BOP from the front led to significant increases in piezo2 expression after 1x (~90%), 2x (~45%), 3x (~80%), and 4x (~88%) exposures. Similarly, exposure to 19 psi BOP from the side caused significantly increase piezo2 after 1x (~64%), 2x (~69%), 3x (~99%), and 4x (~64%) exposures. Piezo2 was also increased after 2x blasts of 16 psi from the front (~55%), but was significantly decreased after 1x (~49%), 3x (~42%), and 4x (~55%) blast exposures of 16 psi from the front. Animals exposed to 16 psi BOP from the side also had a significant increase in piezo2 after 2x (~30%) exposures, but a significant decrease after 1x (~44%), 3 (~67%), and 4x (~46%) blast exposures. Animals exposed to 13 psi BOP from the front had significantly reduced piezo2 expression after 1x (~47%), 3x (~60%), and 4x (~60%) exposures, but no significant change after 2x. Animals exposed to 13 psi BOP from the side also had a significant reduction in piezo2 expression after 1x (~53%), 3x (~52%), and 4x (~63%) exposures, but a significant increase after 2x (~52%) blast exposures (Figure 6).

## Discussion

In this study, we showed that blast overpressure exposure has neuropathological effects which depend upon intensity, orientation, and frequency of blast exposure. BOP exposure can cause alterations in blood-brain barrier structure, as evidenced by changes in the tight junction proteins claudin-5 and occludin as well as in VEGF. A single exposure to blast from the front caused an increase in claudin-5 at all intensities tested, while claudin-5 was significantly decreased after multiple exposures of various intensity and orientation. Occludin expression was also significantly altered in ways that depended on intensity and frequency. VEGF, however, appeared to be most sensitive to relatively lower intensity blast exposure in this study, with significant alterations occurring after 16 and 13 psi blasts. Apart from changes in the BBB, we were interested in investigating other factors that may contribute to pathophysiological changes following blast exposure. TDP-43, which is altered in many neurodegenerative diseases and has been shown to be altered in chronic traumatic encephalopathy (CTE), is also altered following blast exposure. TDP-43 was consistently decreased following 2x blast exposures, independent of intensity, or orientation and seems to be more sensitive to repeated blast exposures. Finally, the mechanosensitive channel protein piezo2, which is involved in the mechanical response to force, is also significantly altered after blast exposure. Piezo2 was very sensitive to intensity, increasing following exposure to 19 psi BOP, and decreasing following exposure to 16 or 13 psi BOP. Together, these data indicate that exposure to BOP causes cerebral vascular and neuronal injury and provide insight into the various aspects of blast that have the most effect on brain injury.

BBB alterations, as evidenced from changes in tight junction proteins and VEGF, may be caused by the mechanical disruption from blast exposure and has been demonstrated after a single BOP exposure and in a few studies with closely-coupled repeated exposures (6, 20–25). In our study, claudin-5 and occludin levels varied depending on blast intensity and number of blast exposures, with most experimental groups showing a decrease in occludin in the brain. Rats exposed to blast from the side orientation seem to have more significant changes in occludin following blast exposure, a phenomenon likely explained by the involvement of lung injury as shown in previous studies, which demonstrate polytrauma following blast (26). This polytrauma can lead to vascular pathology following blast due to alterations in or loss of the cells comprising the BBB such as astrocytes (27, 28) as well as by causing perivascular inflammation (29). Interestingly, similar trends in claudin-5 and occludin data were observed in front exposure groups where an increase was observed in the 1x 19 psi group and a decrease in the 2x 16, 2x 13, 4x 19, and 4x 16 psi groups. Only the frontal exposure 2x 19 psi group shows an increase in occludin but a decrease in claudin-5, while an increase was observed in claudin-5 but not occludin following 3x 16 and 3x 13 psi frontal exposures.

The similarities observed in the front exposure groups were not seen in side exposure groups, demonstrating that orientation affects brain response to blast. One explanation is the effects of lung polytrauma that can alter the brain response (26). Lung injury caused by blast exposure is likely to contribute to injury in the brain, perhaps via a pressure pulse transmitted from the lung to the brain or by contributing to an inflammatory environment in the brain (29–31). Although some investigators report differing changes in intracranial pressure (ICP) after exposure to blast in the front vs. side orientation with a cylindrical shock tube that imparts high dynamic pressure and plateaued, longer duration pressure profiles (32–34), in our model using the ABS, with pressure probes placed in the ventricle and epidural space of the brain, we find no significant differences in ICP at varying orientations (35). Skull shape and structure, which vary in different areas of the head (32), are likely to impart localized strain and stress changes that could potentially contribute to orientation effects that we observed in this study.

Varying vulnerabilities to injurious pressure loads may exist due to neurovascular anatomical relationships in the brain and how they interact with these forces. The orientation of blast exposure results in a complex interplay in forces as the pressure wave propagates through different structures within the brain that may intensify or weaken the force load impacting cerebral tissue depending on head orientation. In particular, heterogeneous neuronal, and glial cytoarchitecture (e.g., orientation of cells in different cortical layers vs. in deep structures such as the thalamus), white matter tract orientation, and regional differences in the vasculature could contribute to differences in pathology based on blast exposure orientation. In front-oriented animals, blast waves would travel anterior-to-posterior in the brain, while in side-oriented animals the whole lateral parasagittal plane of the brain is the first point of blast wave contact. Consequently and as an example, we speculate that the middle cerebral artery, which perfuses the majority of the lateral surface of the cortex, may bear relatively excessive lateral strain when animals are exposed to blast from the side orientation when compared to the front orientation. The microvessels covering the lateral surface of the cortex could be similarly affected and it is conceivable that these vessels could exhibit a greater degree of vulnerability (measured as disruption of BBB proteins). Region specific assessment of the brain for BBB protein changes in tissue fractions can shed further insight into cytoarchitecture. A limitation of this study is that we examined whole hemispheres with Western blot and ELISA, therefore how regional structural differences may have contributed to orientation-dependent neuronal and vascular pathological differences is currently unclear. Future work will further investigate, with techniques such as immunohistochemistry, the effects of blast exposure orientation on BBB disruption.

Another aspect of brain injury mechanisms is acceleration injury, however, we do not observe rotational forces and negligible linear displacement/translation in the blast tube, even without the use of animal restraints. Although orientation could affect localized strain and some pressure differentials, we speculate that the biological changes in the current study could be due to robust differences in lung polytrauma between front and side oriented animals (36). Taken together, irrespective of orientation and frequency, the data show that claudin-5 or occludin are altered in at least one of the treatment groups, demonstrating BBB vulnerability following BOP exposure. While a single exposure led to an increase in claudin-5 or occludin (with the exception of occludin at side exposures to 16 and 19 psi), possibly from compensatory mechanisms from BBB disruption, an opposite response was observed in majority of the repeated exposure groups, demonstrating the brain's vulnerability to repeated exposures. Overall, because there is not a consistent pattern of occludin and claudin-5 expression following blast exposure, exposure intensity and frequency of blast exposure could be important independent influences, and variable cumulative effects are observed with repeated exposure. These BBB proteins exhibit different responses to various combinations of blast orientation, frequency, and intensity.

VEGF was found to be increased mainly in 1x front and side exposure at 13 and 16 psi, where minimal lung injury was observed (<5% of volume) (26). Similar to our results, other studies that have reported an increase in VEGF following blast exposure, with a single exposure or very closely coupled repeated exposures that are >17 psi (37–40). In our study, VEGF was observed to be increased after up to 2x exposures at relatively mild intensity blast (13 psi), which may be more relevant to exposures experienced by breachers. We did not find significant changes in VEGF following 3 or 4 exposures [with the exception of 3x 16 psi (front)]. This might indicate that angiogenesis is activated following 1 or 2 exposures to blast, in order to repair any vascular damage, but returns to baseline and is not further activated after subsequent blasts because the vascular integrity has already been restored. Alternatively, this change in VEGF could be a maladaptive response, which increases vascularization, compromising BBB integrity and leading to damage to neural and glial tissue, a process that is activated following 1 or 2 blast exposures, but is not further activated after subsequent blasts.

In this study, we examined levels of TDP-43 in the whole brain and found significant alterations in TDP-43 after repeated blast, exhibiting the vulnerability of the brain to repeated exposures. TDP-43 is involved in the regulation of thousands of genes, its expression is tightly self-regulated, and alteration in its expression can have many downstream effects (14). It is found to abnormally accumulate (in a similar manner to tau and amyloid-beta) in neuronal and glial protein inclusions, where it is hyperphosphorylated, ubiquitinated, and cleaved, in various neurodegenerative diseases including CTE, frontotemporal lobar degeneration (FTLD), amyotrophic lateral sclerosis (ALS) (14, 15, 41). Under most injury conditions, one exposure did not lead to significant changes in TDP-43 expression. However, we observed a significant reduction in total TDP-43 following 2x blasts, independent of intensity, or orientation. However, significantly increased after 4x 16 psi, regardless of orientation, and 3x 16 psi from the front and 19 psi from the side, demonstrating an increased vulnerability of brain to aggregate pathological proteins when exposed to blast more than 2x. Similarly, it has been found that following brain injury, TDP-43 granules are formed and TDP-43 expression is increased 3 days post injury (42). Complementary to our data, military veterans with a history of blast exposure have been found to show CTE neuropathology with TDP-43 inclusions (15, 43), indicating that blast exposure may lead to TDP-43 proteinopathy. TDP-43 expression is tightly regulated and protein clearance occurs via autophagy and the ubiquitin proteasome system (44). It is speculated that following 2 blast exposures, these clearance mechanisms are activated, leading to the decrease in TDP-43 we observed (Figure 5). We speculate that following > 2 exposures, clearance mechanisms are dysregulated, as has been shown following TBI (45), leading to accumulation of TDP-43 in the brain. These hypotheses are areas of interest for future studies in our group.

Activation of piezo2 by mechanical force such as pressure causes downstream intracellular changes. This neuronal mechanotransduction may play an important role in the cellular injury response seen in TBI (46). Mechanosensitive channels like piezo2 can convey information about the amount of pressure experienced by the channel, and in the brain can convey information about ICP, which increases after blast proportionally to blast intensity and frequency and is accompanied by increased BBB permeability (25). Increased VEGF has also been shown to increase vascular permeability, as well as causing edema and increasing ICP (47, 48). Piezo2 has been shown to change following blast, significantly increasing in the hippocampus of rats exposed to ~14 psi blast at 7 days following BOP exposure, but not following blast of higher (~24 psi) or lower (~10 psi) intensity (49). In our study, the highest intensity tested (19 psi) caused a significant increase in piezo2 expression, irrespective of orientation and repeated exposures, as all experimental groups had about the same amount of piezo2 expression (average 75% increase) where a significant polytrauma was demonstrated (26). Relatively lower BOP exposure showed a generalized decrease in piezo2 following single and repeated exposures (except 2x groups), with minimal polytrauma (as evidenced from <5% lung injury) (26). The 2x exposure groups had increased piezo2 in all intensities and orientations. This finding is reflected in the decreased TDP-43 in all 2x groups, indicating that after 2 exposures, there may be a unique mechanical response in the cell which is reflected by piezo2 expression and that affects protein clearance. Overall, changes in piezo2 expression appear to be dependent on blast intensity more than frequency or orientation, indicating that piezo2 could be a biological dosimeter (reducing the need for physical pressure sensors) for BOP, responding to changes in pressure intensity by increasing expression after high intensity exposure and decreasing expression after lower intensity exposure. This indicates that repeated exposure to lower level, operationally relevant blast may reduce piezo2 in the brain, leading to altered sensitivity to pressure. Future studies are needed to further investigate the findings presented here. One specific area of concern is the time course or longitudinal nature of the injury: are the changes seen after 2, 3, or 4 exposures due to cumulative effects of repeated blast, or due to the time that has elapsed since the first injury? To address this, we must investigate these markers at different time intervals following the last blast exposure, as well as investigate even lower blast intensities that might be more relevant to those in breaching and heavy weapons systems environments.

## Conclusion

This study aimed to fill in research gaps regarding the effects of repetitive low-level blast on the brain, focusing on the BBB, mechanical responses in the brain, and a marker associated with neurodegenerative disease. Piezo2 appears to more sensitive across the spectrum of blast exposure while TDP-43 levels were observed to increase beyond 2x exposures, which could manifest into neurodegenerative pathology. Personnel such as breachers or heavy weapon system operators are exposed to many low-level blasts over an extended period of time while soldiers in combat are more likely to be exposed to fewer, more intense blasts; consequently, it is important to know how intensity and frequency contribute to various negative outcomes. In order to address the complete spectrum of exposures and outcomes, a time-course, region-specific analysis of the brain, along with varying interval of exposures are warranted in future studies.

## Data Availability

All datasets generated for this study are included in the manuscript and/or the Supplementary Files.

## Ethics Statement

All animal experiments were conducted in accordance with the Animal Welfare Act and other federal statutes and regulations relating to animals and experiments involving animals, and adhered to principles stated in the Guide for the Care and Use of Laboratory Animals (NRC Publication 2011 edition) using an Institutional Animal Care and Use Committee approved protocol.

## Author Contributions

LH and VS designed the experiments. LH wrote the manuscript. DW performed the blast experiments. LH, RA, and DW performed data analysis. LH, RA, SG, AB, and RU performed western blot and ELISA experiments. LH, JS, and RA performed statistical analysis. JL, SA, and VS oversaw the study and edited the manuscript.

### Conflict of Interest Statement

VS, LH, JL, and SA have filed a provisional patent application for piezo2 as a biological dosimeter for single and repeated blast exposures. The remaining authors declare that the research was conducted in the absence of any commercial or financial relationships that could be construed as a potential conflict of interest.
