# Supplementary material for: Repeated Low-Level Blast Overpressure Leads to Endovascular Disruption and Alterations in TDP-43 and Piezo2 in a Rat Model of Blast TBI
Source: Front Neurol. 2019 Jul 30;10:766. doi: 10.3389/fneur.2019.00766 (PMC6682625; doi:10.3389/fneur.2019.00766)
Supplement: Supplementary file 1 [file Data_Sheet_1.docx]

Supplementary Material

# Supplementary Figures and Tables

##
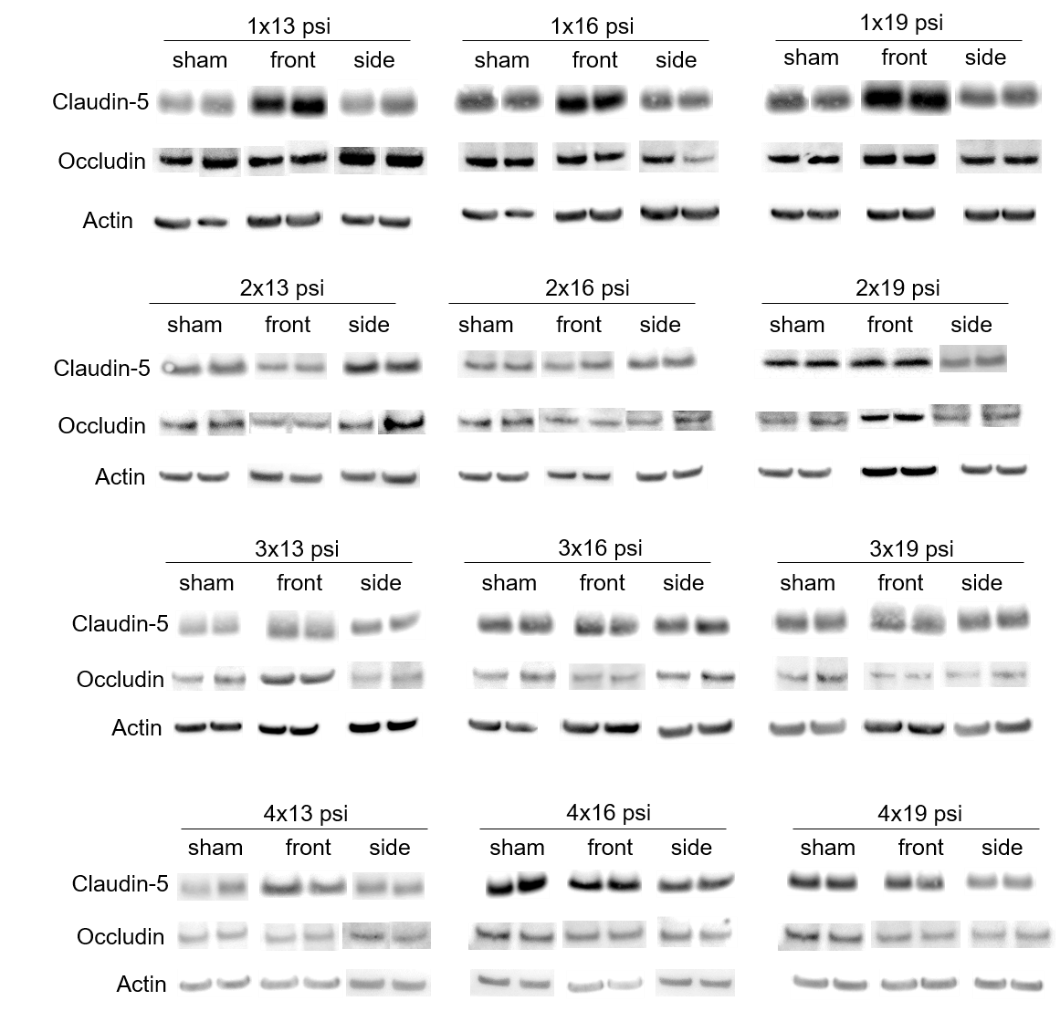
Supplementary Figures

**Supplementary Figure 1.** Representative Western blot images for claudin-5 and occludin, along with β-actin control. Densitometry analysis was used on these blots and normalized to β-actin and experimental groups were normalized to shams. Data is summarized in Figures 3 and 4.

**
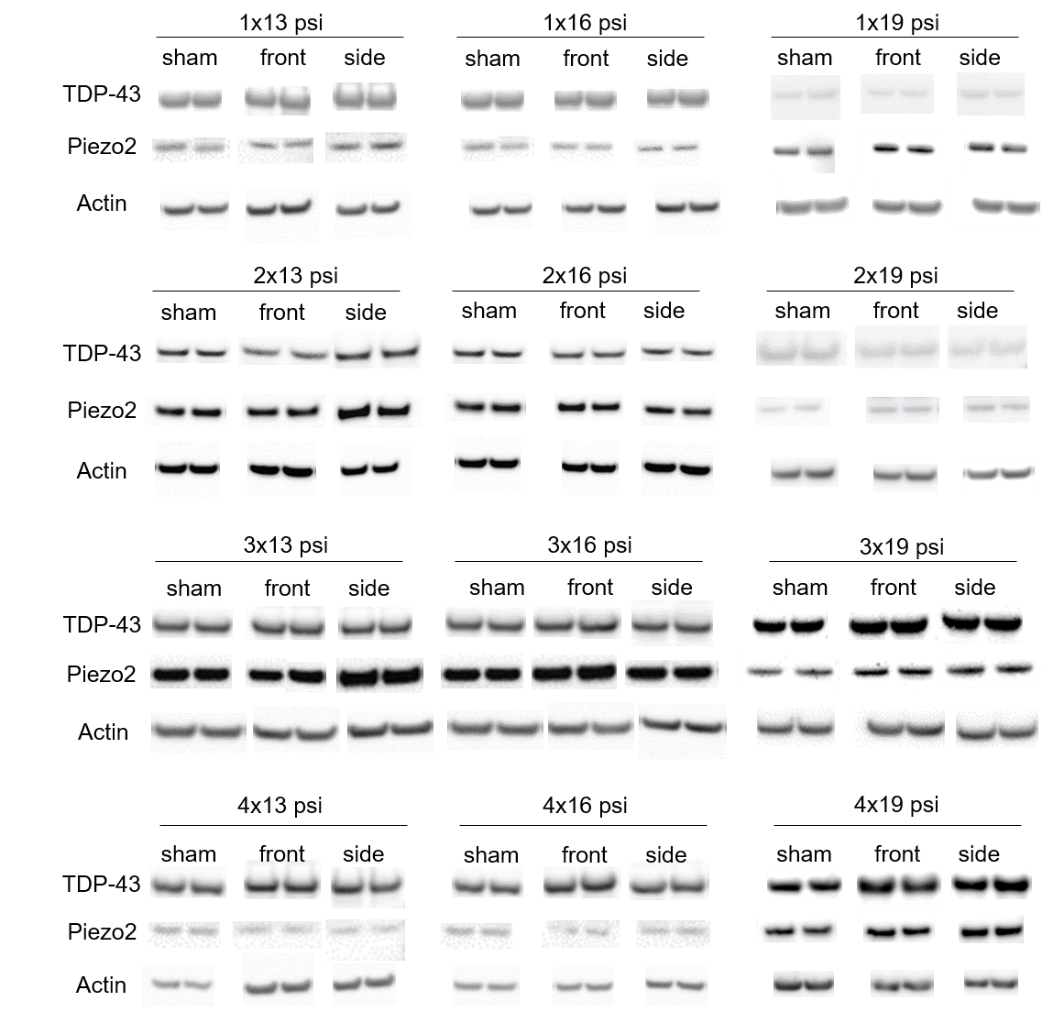
**

**Supplementary Figure 2.** Representative Western blot images for TDP-43 and piezo2, along with β-actin control. Densitometry analysis was used on these blots and normalized to β-actin and experimental groups were normalized to shams. Data is summarized in Figures 5 and 6.

## Supplementary Tables

|  | **19psi (front)** | **19psi (side)** | **16psi (front)** | **16psi (side)** | **13psi (front)** | **13psi (side)** |
| --- | --- | --- | --- | --- | --- | --- |
| **1x blast** | **0.2923** | **0.3958** | **0.0229** | **0.0324** | **0.0131** | **0.0025** |
| **2x blast** | **0.9729** | **0.0324** | **0.4346** | **0.0383** | **0.0191** | **0.0016** |
| **3x blast** | **0.1104** | **0.3958** | **0.0272** | **0.2923** | **0.4756** | **0.7087** |
| **4x blast** | **0.1104** | **0.1264** | **0.2623** | **0.7598** | **0.6588** | **0.9188** |

**Supplementary Table S1**. Significance values for VEGF expression levels determined by Mann-Whitney U test. All values are based on comparison to sham animals.

|  | **19psi (front)** | **19psi (side)** | **16psi (front)** | **16psi (side)** | **13psi (front)** | **13psi (side)** |
| --- | --- | --- | --- | --- | --- | --- |
| **1x blast** | **0.0016** | **0.0137** | **0.3003** | **0.0688** | **0.9777** | **0.8887** |
| **2x blast** | **0.0270** | **0.2511** | **0.0986** | **0.0227** | **0.0047** | **0.0033** |
| **3x blast** | **0.2287** | **0.0861** | **0.6341** | **0.0358** | **0.4353** | **0.0092** |
| **4x blast** | **0.0192** | **0.0173** | **0.0986** | **0.3654** | **0.1701** | **0.0007** |

**Supplementary Table S2**. Significance values for occludin expression levels determined by Mann-Whitney U test. All values are based on comparison to sham animals.

|  | **19psi (front)** | **19psi (side)** | **16psi (front)** | **16psi (side)** | **13psi (front)** | **13psi (side)** |
| --- | --- | --- | --- | --- | --- | --- |
| **1x blast** | **0.0009** | **0.3654** | **0.0066** | **0.9331** | **0.0047** | **0.3855** |
| **2x blast** | **0.0056** | **0.4840** | **0.0056** | **0.0656** | **0.0007** | **0.0108** |
| **3x blast** | **0.6745** | **0.5122** | **0.0607** | **0.1883** | **0.0425** | **0.0778** |
| **4x blast** | **0.0030** | **0.0066** | **0.0358** | **0.5532** | **0.5566** | **0.0688** |

**Supplementary Table S3**. Significance values for claudin-5 expression levels determined by Mann-Whitney U test. All values are based on comparison to sham animals.

|  | **19psi (front)** | **19psi (side)** | **16psi (front)** | **16psi (side)** | **13psi (front)** | **13psi (side)** |
| --- | --- | --- | --- | --- | --- | --- |
| **1x blast** | **0.1106** | **0.3333** | **0.7159** | **0.0270** | **0.5948** | **0.0986** |
| **2x blast** | **0.0033** | **0.0234** | **0.0047** | **0.0066** | **0.0056** | **0.0127** |
| **3x blast** | **1.0000** | **0.0137** | **0.0149** | **0.2511** | **0.7314** | **0.4169** |
| **4x blast** | **0.3333** | **0.3703** | **0.0007** | **0.0115** | **0.5948** | **0.2511** |

**Supplementary Table S4**. Significance values for TDP-43 expression levels determined by Mann-Whitney U test. All values are based on comparison to sham animals.

|  | **19psi (front)** | **19psi (side)** | **16psi (front)** | **16psi (side)** | **13psi (front)** | **13psi (side)** |
| --- | --- | --- | --- | --- | --- | --- |
| **1x blast** | **0.0066** | **0.0163** | **0.0006** | **0.0004** | **0.0007** | **0.0002** |
| **2x blast** | **0.0311** | **0.0234** | **0.0004** | **0.0493** | **0.1701** | **0.0005** |
| **3x blast** | **0.0002** | **0.0007** | **0.0011** | **0.0002** | **0.0007** | **0.0003** |
| **4x blast** | **0.0016** | **0.0009** | **0.0007** | **0.0008** | **0.0002** | **0.0002** |

**Supplementary Table S5**. Significance values for piezo2 expression levels determined by Mann-Whitney U test. All values are based on comparison to sham animals.
